# Supplementary figures and images for: Overlap matrix completion for predicting drug-associated indications
Source: PLoS Comput Biol. 2019 Dec 23;15(12):e1007541. doi: 10.1371/journal.pcbi.1007541 (PMC6946175; doi:10.1371/journal.pcbi.1007541)

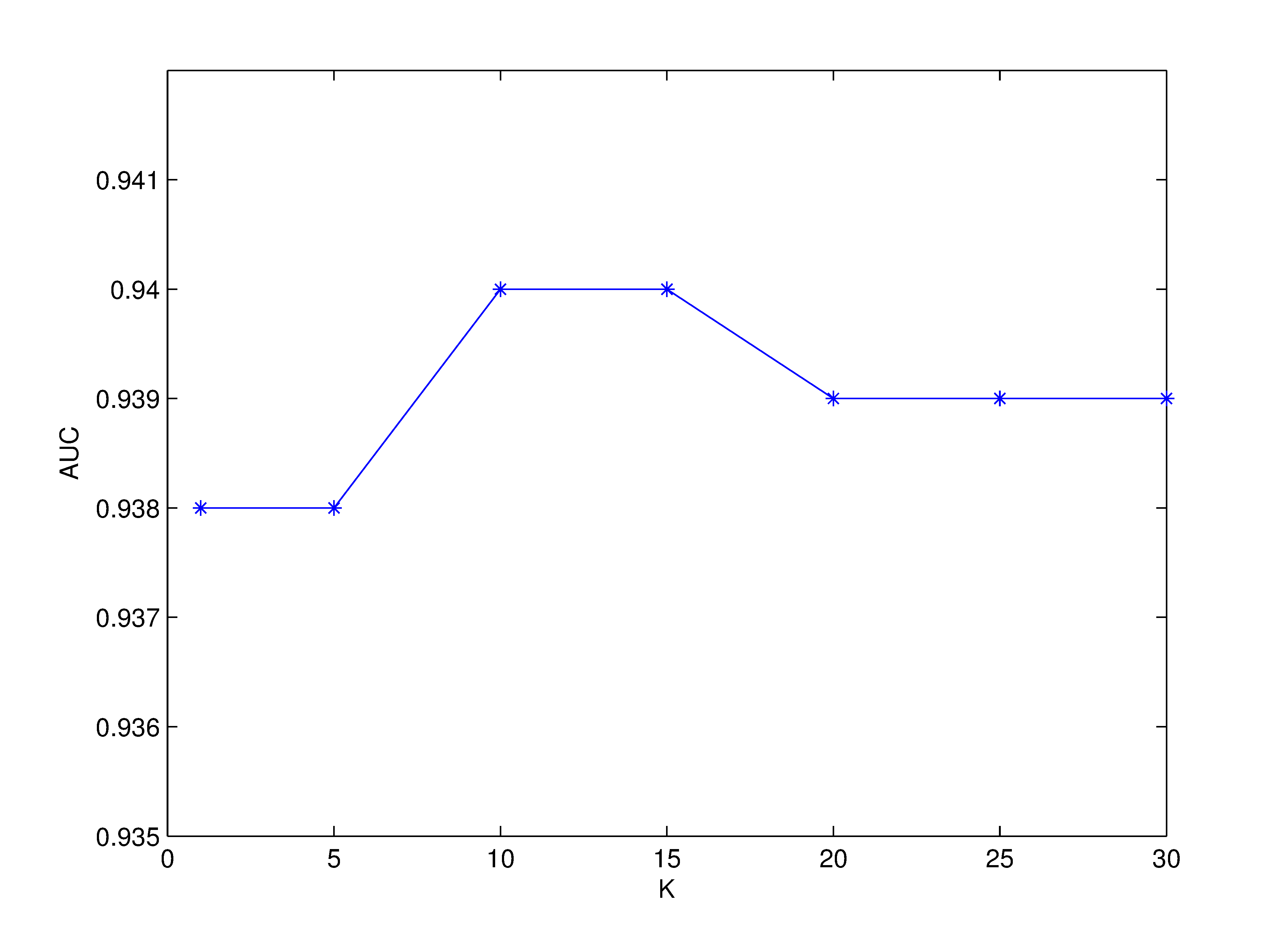

Supplement: S1 Fig — (TIF) [file pcbi.1007541.s001.tif]

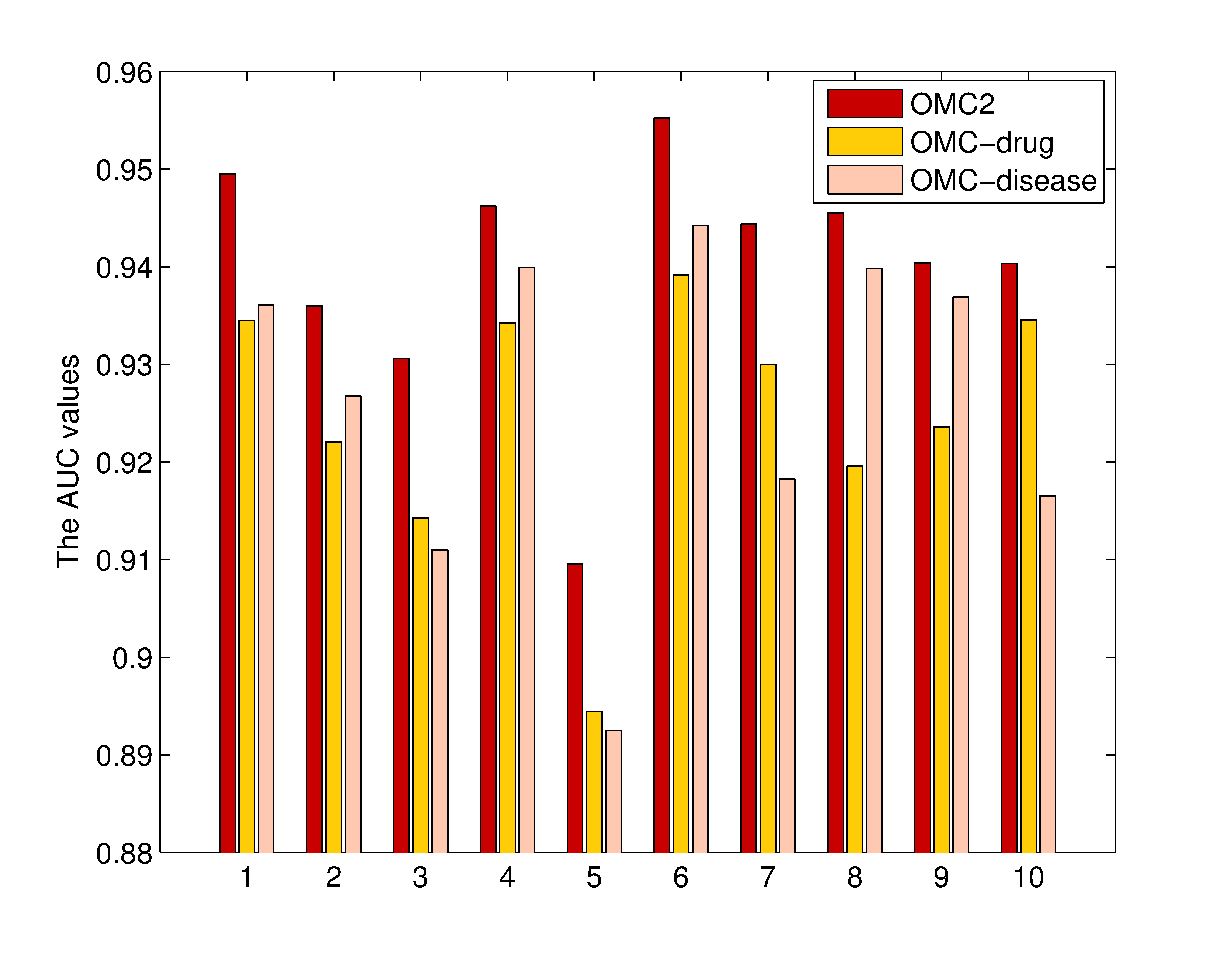

Supplement: S2 Fig — The result of each fold is presented. (TIF) [file pcbi.1007541.s002.tif]

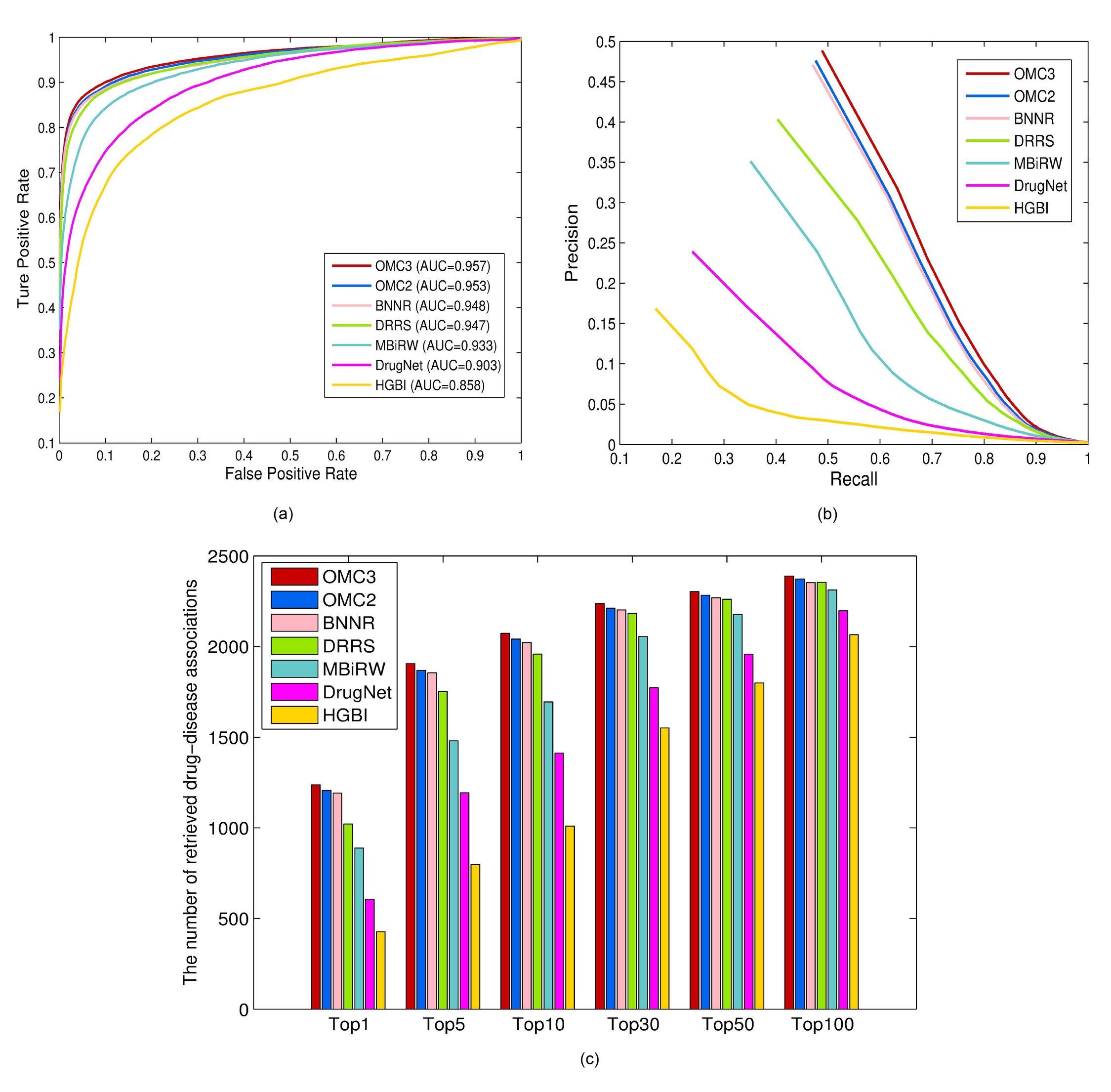

Supplement: S3 Fig — (a) ROC curves of prediction results. (b) PR curves of predicting candidate diseases for drugs. (c) The number of correctly retrieved drug–disease associations for various rank thresholds. The performance of all methods for predicting drug–disease associations in the 10-fold cross-validation on CDataset. (TIF) [file pcbi.1007541.s003.tif]

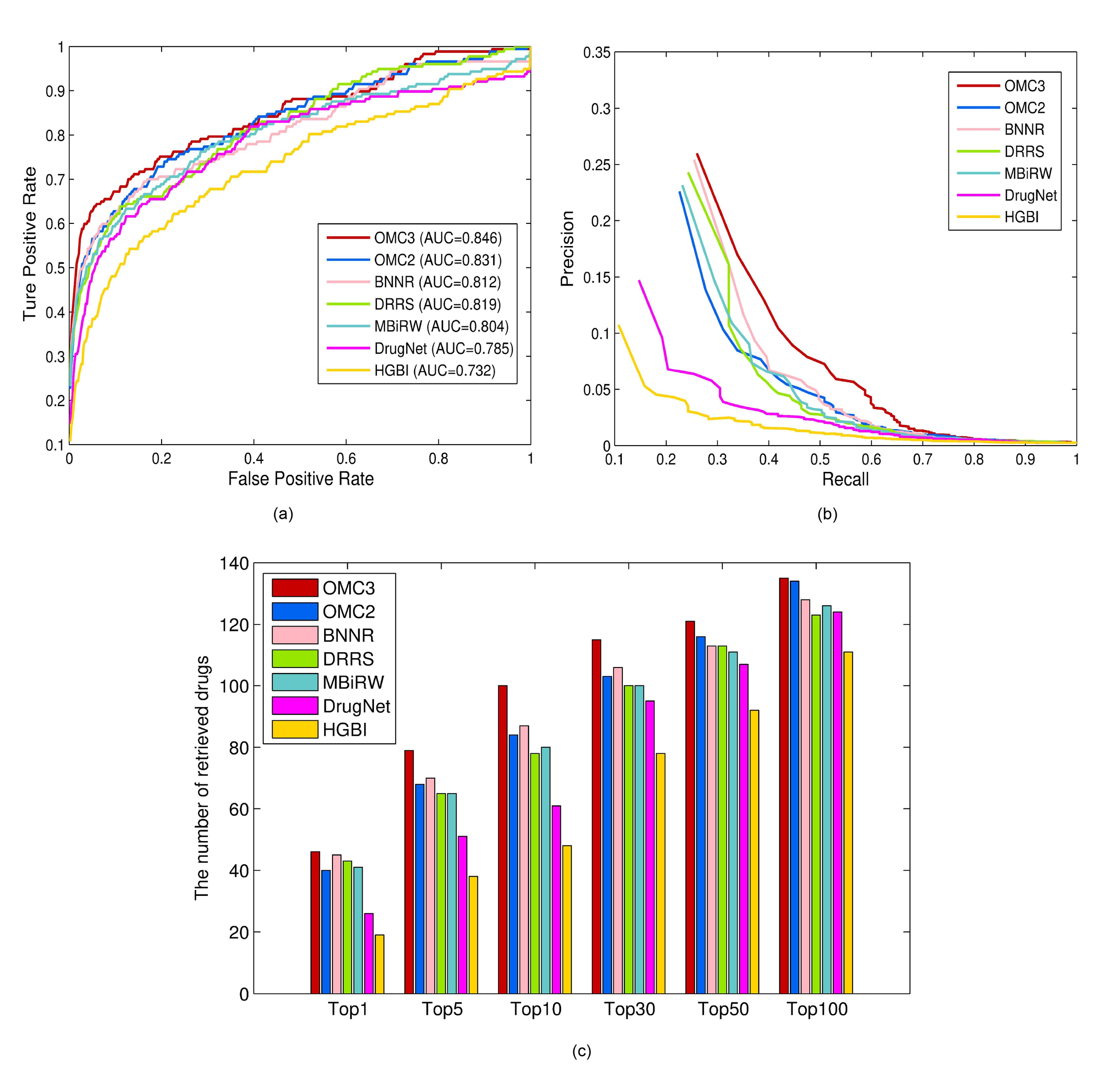

Supplement: S4 Fig — (a) ROC curves of prediction results. (b) PR curves of predicting candidate diseases for drugs. (c) The number of correctly retrieved drug–disease associations for various rank threshold. The performance of all methods in predicting potential diseases for new drugs on CDataset. (TIF) [file pcbi.1007541.s004.tif]

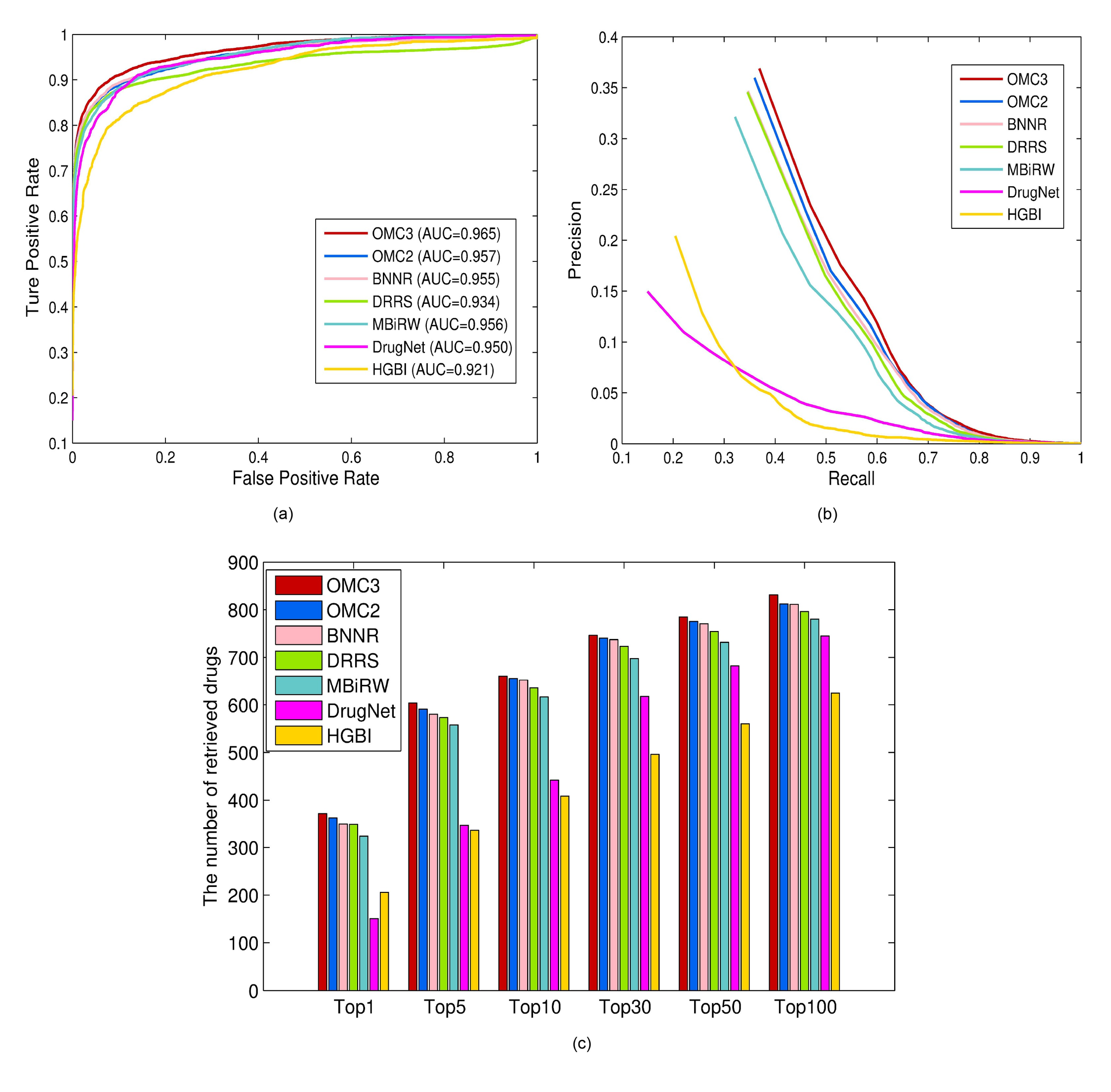

Supplement: S5 Fig — (a) ROC curves of prediction results. (b) PR curves of predicting candidate diseases for drugs. (c) The number of correctly retrieved drug–disease associations for various rank thresholds. The performance of all methods for predicting drug–disease associations in the 10-fold cross-validation on DNdataset. (TIF) [file pcbi.1007541.s005.tif]

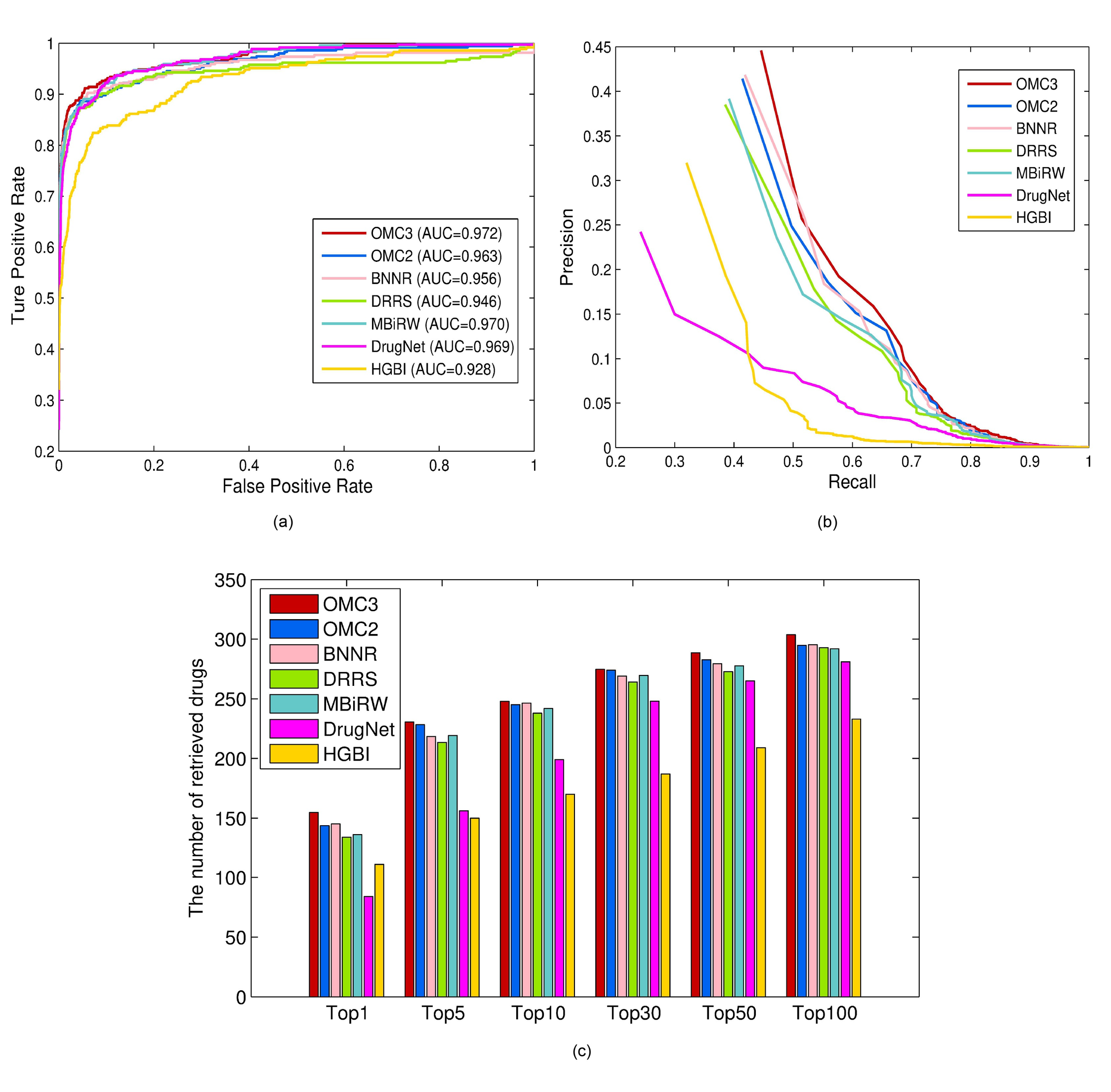

Supplement: S6 Fig — (a) ROC curves of prediction results. (b) PR curves of predicting candidate diseases for drugs. (c) The number of correctly retrieved drug–disease associations for various rank threshold. The performance of all methods in predicting potential diseases for new drugs on DNdataset. (TIF) [file pcbi.1007541.s006.tif]
