# Supplementary material for: Overlap matrix completion for predicting drug-associated indications
Source: PLoS Comput Biol. 2019 Dec 23;15(12):e1007541. doi: 10.1371/journal.pcbi.1007541 (PMC6946175; doi:10.1371/journal.pcbi.1007541)
Supplement: S1 Table — (DOC) [file pcbi.1007541.s007.doc]

**S1 Table.** The AUC values under different values of $\alpha$ and $\beta$ in the 10-fold cross-validation for the gold standard dataset.

| $\alpha\backslash\beta$ | 0.1 | 1 | 10 | 100 |
| --- | --- | --- | --- | --- |
| 0.1 | 0.778 | 0.786 | 0.872 | 0.911 |
| 1 | 0.898 | 0.933 | **0.938** | 0.917 |
| 10 | 0.912 | 0.937 | 0.937 | 0.915 |
| 100 | 0.913 | 0.937 | 0.937 | 0.915 |

The best AUC result in this table is **bold**.
