# Supplementary material for: Overlap matrix completion for predicting drug-associated indications
Source: PLoS Comput Biol. 2019 Dec 23;15(12):e1007541. doi: 10.1371/journal.pcbi.1007541 (PMC6946175; doi:10.1371/journal.pcbi.1007541)
Supplement: S2 Table — (DOC) [file pcbi.1007541.s008.doc]

**S2 Table.** The AUC values based on K=10 for $\alpha$and $\beta$ in the 10-fold cross-validation for the gold standard dataset.

| $\alpha\backslash\beta$ | 0.1 | 1 | 10 | 100 |
| --- | --- | --- | --- | --- |
| 0.1 | 0.779 | 0.789 | 0.871 | 0.918 |
| 1 | 0.887 | 0.928 | **0.940** | 0.918 |
| 10 | 0.902 | 0.933 | 0.939 | 0.916 |
| 100 | 0.903 | 0.933 | 0.939 | 0.915 |

The best AUC result in this table is **bold**.
