# Supplementary material for: Overlap matrix completion for predicting drug-associated indications
Source: PLoS Comput Biol. 2019 Dec 23;15(12):e1007541. doi: 10.1371/journal.pcbi.1007541 (PMC6946175; doi:10.1371/journal.pcbi.1007541)
Supplement: Attachment — Submitted filename: Response to reviewers.doc [file pcbi.1007541.s010.doc]

**Responses to the Review Comments**

On the paper

**"**Overlap matrix completion for predicting drug-associated indications**"**

By Mengyun Yang, Huimin Luo, Yaohang Li, Fang-Xiang Wu, and Jianxin Wang.

Submitted to PLOS Computational Biology [PCOMPBIOL-D-19-01173]

Dear the editors and reviewers,

We would like to thank you for your careful reading, helpful comments, and constructive suggestions, which have significantly improved the presentation of our manuscript.

We have carefully considered all comments from the reviewers and revised our manuscript accordingly. The manuscript has also been double-checked by proficient English speakers, and the typos and grammar errors we found have been corrected. The revised parts are highlighted with yellow color in the revised manuscript. In the following section, we summarize our responses to each comment from the reviewers. We believe that our responses have well addressed all concerns from the reviewers. We hope our revised manuscript can be accepted for publication.

Sincerely,

Jianxin Wang

Professor, School of Computer Science and Engineering

Central South University

Changsha, Hunan P.R. China

Email: [jxwang@mail.csu.edu.cn](mailto:jxwang@mail.csu.edu.cn)

**Answers to Reviewer 1**

*Comments:*

*This paper formulates computational drug repositioning problem based on matrix completion. An overlap matrix completion method is proposed to predict the potential drug-associated indications by integrating reliable drug-related and disease-related information in bilayer and tri-layer networks. Experimental studies on three different datasets show that the proposed approach can outperform several baseline methods, especially in tri-layer networks.*

**Author’s Response:**

We thank the reviewer for reading our paper carefully and giving the above positive comments.

*Detailed comments:*

1. *When I take a look at case studies, only three drugs are used as examples. In order to give more references for medical researchers, the predicted top-30 indications of each drug need to be listed in the supplementary file.*

**Author’s Response:**

We agree with the reviewer that listing the predicted top-30 indications of each drug will give helpful references for medical researchers. We have added them to Supporting Information (the supplementary file).

*2. Could MBiRW algorithm be used for comparison in tri-layer networks? Please clarify it.*

**Author’s Response:**

Thank you for the above comment. There are two main reasons that we have not used MBiRW in the tri-layer networks for comparison: Firstly, the comprehensive similarity measures of MBiRW are constructed between two kinds of biological entities, and it is not suitable for handling correlation information among three biological entities simultaneously. For instance, MBiRW calculates the comprehensive drug similarity by combining drug characteristics and drug-disease associations, rather than combining multiple association information at the same time. Secondly, a corresponding tri-layer model of MBiRW has not been proposed yet. If we simply replace MBiRW with Random Walk algorithm in the tri-layer networks, it would not truly evaluate the performance of MBiRW algorithm. Therefore, we don’t present the MBiRW algorithm in tri-layer networks.

*3. The first letter of 'hyperplastic' should be capitalized in Table 2.*

**Author’s Response:**

Thank you for pointing out this mistake in our paper. We have corrected it in the revised manuscript.

*4. There are some writing and grammatical errors in the manuscript. The author should examine and revise the article carefully.*

**Author’s Response:**

We thank the reviewer for reading our paper carefully. We have thoroughly checked and corrected all grammatical errors and typos we found in our revised manuscript.

*5. The quality of pictures in the manuscript need to be improved.*

**Author’s Response:**

Thank you for the above suggestions. We have redrawn Fig 1 and Fig 2 by adjusting the appropriate size and enlarging the graph. In addition, we have used the Preflight Analysis and Conversion Engine (PACE) to ensure all figures meet PLOS requirements.

*6．(1) Drug combination can be regarded as a new way of drug repositioning. Could you give some discussions whether your method could be used for synergistic drug combination prediction in the future (PMID: 27415801 should be discussed)?* **Author’s Response:**

Thank you for the above comments and suggestions. The synergistic drug combination is based on the assumption that principal drugs which obtain synergistic effect with similar adjuvant drugs are often similar and vice versa [1]. That means the drug combination matrix is low-rank. Therefore, the overlapped matrix completion can predict potential synergistic drug combination by integrating drug similarity matrix and drug-target interaction matrix. In addition, it may avoid classifying into principal drug and adjuvant drug before obtaining the final score of drug combination.

(2) *In addition, could you consider the possibility of developing computational models for drug repositioning based on drug related miRNA, a new kind of drug target? Some important studies should be discussed (PMIDs: 30325405 and 29943160).*

**Author’s Response:**

Thanks for the above comments and suggestions. MiRNAs play an important role in the initiation and development of various human diseases. Several drug-like compound libraries targeting different miRNAs were successfully screened in cell assays, further demonstrating the possibility of targeting miRNAs with small molecules. Hence, developing computational models for drug repositioning based on drug related miRNA is very meaningful and promising. Some original and novel methods have been proposed in recent years [2]. Especially, based on tri-layer heterogeneous, more prior information has been used to obtain better prediction performance [3]. In the future, we will use matrix completion method to explore drug combination and miRNA-small molecule association for drug repositioning.

In the section of Conclusion in the revised manuscript, we have added some discussion about synergistic drug combination prediction and drug repositioning based on drug related miRNA.

[1]Chen X, Ren B, Chen M, Wang Q, Zhang L, and Yan G. NLLSS: Predicting Synergistic Drug Combinations Based on Semi-supervised Learning. PLOS Computational Biology. 2016; 12(7): e1004975.

[2]Chen X, Guan NN, Sun YZ, Li JQ, and Qu J. MicroRNA-small molecule association identification: from experimental results to computational models. Briefings in Bioinformatics. 2018; 16: 1–15.

[3]Qu J, Chen X, Sun Y, Zhou L, Jian Q, and Ming Z. Inferring potential small molecule-miRNA association based on triple layer heterogeneous network. Journal of Cheminformatics. 2018; 10(1): 30.

*7. Please analyze the novelty and limitations of the method in the section of Conclusions.*

**Author’s Response:**

Thank you for the above suggestion. We have added the analysis to paragraphs of conclusions to discuss the novelty and limitations of the overlapped matrix completion in the section of Conclusion.

**Answers to Reviewer 2**

*Comments:*

*This manuscript proposed a new method, namely the overlap matrix completion (OMP) method, to find potential drug-associated relationship. Numerical results show that the new algorithm can achieve accurate results with high efficiency. This is a well-written and well-organized work to address an important issue in computational biology.*

**Author’s Response:**

We thank the reviewer for reading our manuscript and giving the above positive comments.

*Detailed issues:*

1. *The computation to find the optimal values of alpha, beta and K may not be optimal. First the samples are not evenly distributed, for example, alpha \in [0.1. 1 10, 100]. It may be possible the optimal value is actually around 2 or around 5.*

**Author’s Response:**

Thank you for the above comments and suggestions. The optimal values of $\alpha$ and $\beta$ may be around 2 or 5, because the search range of the interval is more precise. We perform a cross validation computation to determine them by the grid search, which are chosen from$\left\{ 2^{-2}，2^{-1}，2^{0}，2^{1}，2^{2}，2^{3}，2^{4}，2^{5}，2^{6} \right\}$ or $\left\{ 5^{-2}，5^{-1}，5^{0}，5^{1}，5^{2}，5^{3} \right\}$, respectively. When the neighborhood size K is fixed to 1, the AUC values are listed in Table 1 and Table 2, respectively. As shown in Table 1 and Table 2, the best AUC values are the same and are equal to 0.941. Considering the search efficiency, we choose $\left\{ 5^{-2}，5^{-1}，5^{0}，5^{1}，5^{2}，5^{3} \right\}$ as the possible parameter values.

In Table 2, there are three options to achieve the best AUC value. In this case, we use the precision as the second evaluation metric. When $\beta$ is equal to $5^{1}$, the best value of precision is 0.438 where $\alpha$ is equal to $5^{2}$. Therefore, our result shows that the best performance is achieved by $\alpha=\text{25}$ and $\beta=5$.

**Table 1.** The AUC values under $\alpha, \beta\in\left\{ 2^{-2}，2^{-1}，2^{0}，2^{1}，2^{2}，2^{3}，2^{4}，2^{5}，2^{6} \right\}$ in the 10-fold cross-validation for the gold standard dataset.

| $\alpha$ | $2^{-2}$ | $2^{-1}$ | $2^{0}$ | $2^{1}$ | $2^{2}$ | $2^{3}$ | $2^{4}$ | $2^{5}$ | $2^{6}$ |
| --- | --- | --- | --- | --- | --- | --- | --- | --- | --- |
| $2^{-2}$ | 0.836 | 0.852 | 0.869 | 0.895 | 0.917 | 0.928 | 0.934 | 0.935 | 0.929 |
| $2^{-1}$ | 0.889 | 0.903 | 0.918 | 0.931 | 0.937 | 0.938 | 0.936 | 0.933 | 0.925 |
| $2^{0}$ | 0.914 | 0.925 | 0.933 | 0.938 | 0.940 | 0.939 | 0.935 | 0.928 | 0.922 |
| $2^{1}$ | 0.922 | 0.930 | 0.936 | 0.940 | **0.941** | 0.939 | 0.933 | 0.924 | 0.919 |
| $2^{2}$ | 0.924 | 0.931 | 0.937 | 0.940 | **0.941** | 0.939 | 0.933 | 0.922 | 0.917 |
| $2^{3}$ | 0.924 | 0.932 | 0.937 | 0.940 | **0.941** | 0.939 | 0.932 | 0.920 | 0.916 |
| $2^{4}$ | 0.924 | 0.932 | 0.937 | 0.940 | **0.941** | 0.939 | 0.933 | 0.919 | 0.915 |
| $2^{5}$ | 0.924 | 0.932 | 0.937 | 0.941 | **0.941** | 0.938 | 0.933 | 0.918 | 0.915 |
| $2^{6}$ | 0.924 | 0.932 | 0.937 | 0.941 | **0.941** | 0.939 | 0.933 | 0.917 | 0.915 |

The best AUC results in this table are **bold**.

**Table 2.** The AUC values under $\alpha,\beta\in\left\{ 5^{-2}，5^{-1}，5^{0}，5^{1}，5^{2}，5^{3} \right\}$ in the 10-fold

cross-validation for the gold standard dataset.

| $\alpha$ | $5^{-2}$ | $5^{-1}$ | $5^{0}$ | $5^{1}$ | $5^{2}$ | $5^{3}$ |
| --- | --- | --- | --- | --- | --- | --- |
| $5^{-2}$ | 0.678 | 0.749 | 0.756 | 0.776 | 0.911 | 0.906 |
| $5^{-1}$ | 0.814 | 0.821 | 0.847 | 0.907 | 0.935 | 0.901 |
| $5^{0}$ | 0.882 | 0.911 | 0.933 | 0.940 | 0.931 | 0.906 |
| $5^{1}$ | 0.895 | 0.921 | 0.937 | **0.941** | 0.926 | 0.915 |
| $5^{2}$ | 0.895 | 0.922 | 0.937 | **0.941** | 0.926 | 0.915 |
| $5^{3}$ | 0.895 | 0.922 | 0.937 | **0.941** | 0.926 | 0.915 |

The best AUC results in this table are **bold**.

For the parameter K, we assign 25 and 5 to $\alpha$ and $\beta$, respectively, and use the cross validation to pick an appropriate K from {1,5,10,15,20,25,30}. Fig 1 shows the corresponding AUC values under this setting. When the neighborhood size K is 10, the best AUC value is achieved.

Fig 1. The plot of AUC values is indicated by the OMC2 algorithm when the neighborhood size K is chosen from {1,5,10,15,20,25,30} in cross-validation.

Compared with the parameter selection $\alpha=1， \beta=\text{10}， K=\text{10}$ (so-called PS1) for OMC2 and OMC3, we conduct the same 10-fold cross-validation and *denovo* test under the parameter selection $\alpha=\text{25}， \beta=5， K=\text{10}$ (so-called PS2). As shown in Table 3, PS1 can lead to better performance in the *de novo* test while PS2 performs slightly better in the 10-fold cross-validation. In addition, PS1 can obtain the optimal values of parameters faster, because it only needs to pick the optimal parameters from 16 combinatorial schemes.

According to efficiency and performance, we finally choose $\alpha=1， \beta=\text{10}， K=\text{10}$as the optimal parameters.

**Table 3.** The AUC values of OMC2 and OMC3 under two different parameter selections in the 10-fold cross-validation and *de novo* test, respectively.

| AUC | OMC2 | | OMC3 | |
| --- | --- | --- | --- | --- |
|  | 10-fold cross-validation | *De novo* | 10-fold cross-validation | *De novo* |
| $\alpha=1， \beta=\text{10}， K=\text{10}$  (PS1) | 0.939 | **0.851** | 0.945 | **0.872** |
| $\alpha=\text{25}， \beta=5， K=\text{10}$  (PS2) | **0.940** | 0.845 | **0.946** | 0.866 |

The best AUC results in this table are **bold**.

2. *In addition, the work first fixed K=1 and found the optimal value of alpha=1, beta=10. Then based on alpha=1, beta=10, found the optimal value was K=10. The question is, if K=10, what is the optimal values of alpha and beta?*

**Author’s Response:**

Thank you for the above comments. We have fixed the neighborhood size K to 10, and the optimal values of $\alpha$ and $\beta$ are also equal to 1 and 10, respectively. The results are listed in Table 4 and it could further illustrate the stability of the parameter values.

Table 4. The AUC values based on K=10 for $\alpha$and $\beta$ in the 10-fold cross-validation.

| $\alpha$ | 0.1 | 1 | 10 | 100 |
| --- | --- | --- | --- | --- |
| 0.1 | 0.779 | 0.789 | 0.871 | 0.918 |
| 1 | 0.887 | 0.928 | **0.940** | 0.918 |
| 10 | 0.902 | 0.933 | 0.939 | 0.916 |
| 100 | 0.903 | 0.933 | 0.939 | 0.915 |

The best AUC result in this table is **bold**.

In the section of Parameter settings, we have added a description reflecting the above comment.

3. *Is there any possibility to develop an iterated scheme to search for the optimal values?*

**Author’s Response:**

Thank you for the above comment. Choosing the optimal values of the parameters is a challenging problem, and the general scheme is the grid search. However, an adaptive penalty scheme [1] for $\beta$ has been proposed inrecent years. The adaptive updating strategy for the penalty parameter is described as follows,

$$\beta_{k+1}=\text{min}(\beta_{\text{max}},\text{ρβ}_{k})$$

where $\beta_{\text{max}}=\text{10}^{\text{20}},\rho=1\text{.}\text{05},\beta_{0}=\text{10}^{-4}$.

We used this adaptive penalty scheme to search for the optimal parameters ($\alpha$and *K*) of OMC, which means the scheme can reduce one parameter ($\beta$). $\alpha$ is chosen from $\left\{ 5^{-2}，5^{-1}，5^{0}，5^{1}，5^{2}，5^{3} \right\}$ and *K* is from $\left\{ 1，5，\text{10}，\text{15},\text{20}，\text{25},\text{30} \right\}$. Table 5 shows the AUC values of OMC2 under different values of $\alpha$ and *K* on the gold standard dataset. However, the best AUC is only 0.920, which is smaller than the best AUC value of PS1 (AUC=0.938). Therefore, the adaptive penalty scheme is not reported in this study.

**Table 5.** The AUC values under $\alpha\in\left\{ 5^{-2}，5^{-1}，5^{0}，5^{1}，5^{2}，5^{3} \right\}$ and $K\in\left\{ 1，5，\text{10}，\text{15},\text{20}，\text{25},\text{30} \right\}$ in the 10-fold cross-validation for the gold standard dataset.

| $\alpha$ | 1 | 5 | 10 | 15 | 20 | 25 | 30 |
| --- | --- | --- | --- | --- | --- | --- | --- |
| $5^{-2}$ | 0.762 | 0.766 | 0.766 | 0.766 | 0.766 | 0.766 | 0.765 |
| $5^{-1}$ | 0.820 | 0.819 | 0.817 | 0.815 | 0.814 | 0.813 | 0.812 |
| $5^{0}$ | 0.908 | 0.902 | 0.899 | 0.897 | 0.895 | 0.895 | 0.894 |
| $5^{1}$ | 0.919 | 0.913 | 0.910 | 0.909 | 0.908 | 0.907 | 0.907 |
| $5^{2}$ | **0.920** | 0.914 | 0.911 | 0.909 | 0.908 | 0.908 | 0.907 |
| $5^{3}$ | **0.920** | 0.914 | 0.911 | 0.909 | 0.908 | 0.908 | 0.907 |

The best AUC results in this table are **bold**.

[1]Shang F, Cheng J, Liu Y, Luo ZQ, Lin Z. Bilinear Factor Matrix Norm Minimization for Robust PCA: Algorithms and Applications. IEEE Transactions on Pattern Analysis and Machine Intelligence, 2017:1-1.

4. *There are many curves in each figure, and 11 figures in this work. It may be better to put some figures in supplementary information and show the remaining figures in larger size.*

**Author’s Response:**

Thank you for the above suggestions. Some major figures, including Fig 1, Fig 2, Fig 4, Fig 5, and Fig 7 in original manuscript, have been shown in the larger size. The remaining figures have been moved to Supporting Information.
